# Genome-wide copy number variation (CNV) detection in Nelore cattle reveals highly frequent variants in genome regions harboring QTLs affecting production traits

**DOI:** 10.1186/s12864-016-2752-9

**Published:** 2016-06-13

**Authors:** Joaquim Manoel da Silva, Poliana Fernanda Giachetto, Luiz Otávio da Silva, Leandro Carrijo Cintra, Samuel Rezende Paiva, Michel Eduardo Beleza Yamagishi, Alexandre Rodrigues Caetano

**Affiliations:** Faculdade de Ciências Agrárias, Biológicas e Sociais Aplicadas, Universidade do Estado de Mato Grosso (UNEMAT), Av. Prof Dr. Renato Figueiro Varella, CEP 78.690-000 Nova Xavantina, Mato Grosso Brazil; Programa de Pós-Graduação em Genética e Biologia Molecular–Instituto de Biologia, Universidade Estadual de Campinas (UNICAMP), Campinas, São Paulo Brazil; Embrapa Informática Agropecuária - Laboratório Multiusuário de Bioinformática (LMB), Campinas, São Paulo Brazil; Embrapa Gado de Corte, Campo Grande, Mato Grosso do Sul Brazil; Embrapa – Secretaria de Relações Internacionais, Brasília, Distrito Federal Brazil; Embrapa Recursos Genéticos e Biotecnologia, Brasília, Distrito Federal Brazil; CNPq Fellow, ᅟ, ᅟ

**Keywords:** Beef cattle, Copy number variants, High-throughput nucleotide sequencing, SNP genotyping

## Abstract

**Background:**

Copy number variations (CNVs) have been shown to account for substantial portions of observed genomic variation and have been associated with qualitative and quantitative traits and the onset of disease in a number of species. Information from high-resolution studies to detect, characterize and estimate population-specific variant frequencies will facilitate the incorporation of CNVs in genomic studies to identify genes affecting traits of importance.

**Results:**

Genome-wide CNVs were detected in high-density single nucleotide polymorphism (SNP) genotyping data from 1,717 Nelore (Bos indicus) cattle, and in NGS data from eight key ancestral bulls. A total of 68,007 and 12,786 distinct CNVs were observed, respectively. Cross-comparisons of results obtained for the eight resequenced animals revealed that 92 % of the CNVs were observed in both datasets, while 62 % of all detected CNVs were observed to overlap with previously validated cattle copy number variant regions (CNVRs). Observed CNVs were used for obtaining breed-specific CNV frequencies and identification of CNVRs, which were subsequently used for gene annotation. A total of 688 of the detected CNVRs were observed to overlap with 286 non-redundant QTLs associated with important production traits in cattle. All of 34 CNVs previously reported to be associated with milk production traits in Holsteins were also observed in Nelore cattle. Comparisons of estimated frequencies of these CNVs in the two breeds revealed 14, 13, 6 and 14 regions in high (>20 %), low (<20 %) and divergent (NEL > HOL, NEL < HOL) frequencies, respectively.

**Conclusions:**

Obtained results significantly enriched the bovine CNV map and enabled the identification of variants that are potentially associated with traits under selection in Nelore cattle, particularly in genome regions harboring QTLs affecting production traits.

**Electronic supplementary material:**

The online version of this article (doi:10.1186/s12864-016-2752-9) contains supplementary material, which is available to authorized users.

## Background

Copy number variations (CNVs) have been shown to account for substantial portions of genomic variation in humans. Gains or losses in genomic regions varying from 50 bp to several megabases (Mbp) in size have been estimated to cover 77.97 % of the human genome (http://dgv.tcag.ca/dgv/app/statistics?ref=GRCh37/hg19) [1]. CNVs have also been shown to cause changes in transcription levels of specific genes and may be an important source of material for evolutionary mechanisms to act upon [2]. Approximately half of observed human CNVs span regions containing protein-coding genes [1] known to be involved in essential cellular functions, general metabolism and the onset of different diseases [3–9], and which may influence disease susceptibility [10–12]. CNV alterations have also been observed in primary and metastatic cancerous tissues [4, 11, 13–15] and to be associated with various genetic traits [11, 16].

Most reported broad population-oriented studies for CNV detection use at least two main platforms: Comparative Genomic Hybridization (CGH) arrays and SNP genotyping arrays [17–19]. Advantages and disadvantages associated with these platforms have been widely discussed in the literature [20–22]. However, with the advent and rapidly decreasing costs of next generation sequencing (NGS), studying CNVs with sequencing data has also become increasingly feasible [23, 24]. The main advantages of sequencing over genotyping lie in the improved resolution of CNV identification, and particularly in the fact that searches for CNVs are not limited to specific, pre-defined regions. NGS protocols randomly generate reads and therefore close to the entire genome can be sampled with high coverage and resolution, thus promoting higher accuracy in CNV detection and greater precision when estimating breakpoints [24, 25].

Studies to identify and catalogue CNVs have been successfully performed on animals of economic importance, including catlle [26–37], chicken [38, 39], pig [40, 41], sheep [42, 43] and goat [44]. A large number of CNVs were identified in taurine (B*os taurus)* and zebuine cattle (*Bos indicus*) in regions containing genes known to affect complex traits [17, 18, 26, 29, 31, 32]. The overlap of CNVs reported among animals of different taurine breeds is greater than the overlap between taurine and indicine cattle while, even though analyses were performed with data from a single Nelore (*B. indicus*) sample, zebu cattle were observed to have the largest CNV diversity among studied breeds [26].

The present study is the first to widely and deeply analyze a population of Nelore (Zebu) cattle composed of 1,717 animals that were genotyped at high density (~770 K SNPs). In addition, eight key ancestral bulls were resequenced with minimal coverage of 20×. The goal of this study was to perform a high-resolution analysis to detect and characterize CNVs in this breed while also estimating breed-specific variant frequencies.

## Results and discussion

### Genome-wide discovery and distribution of CNVs

**Fig. 1:**
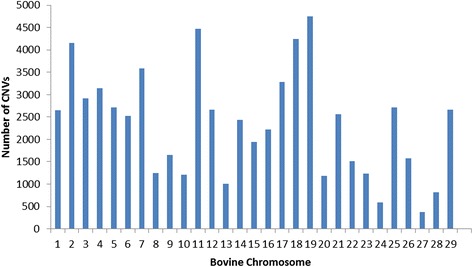
Chromosome distribution of CNVs detected with high-density SNP genotyping data from Nelore cattle

A total of 68,007 CNVs representing 54,510 single copy duplications, 1,729 double copy duplications, 11,672 single copy deletions, and 96 double copy deletions (Additional file 1) were detected with the analysis of genotyping data from 1,509 Nelore samples which passed data QC procedures. Figure 1 shows the chromosome distribution of all detected CNVs. A total of 1,411, 515, and 24 CNVs were observed in >1, >2 and >10 % of the samples analyzed, respectively.

**Fig. 2:**
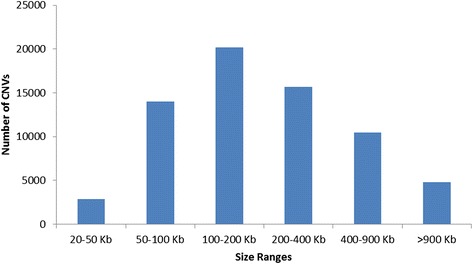
Size distribution of CNVs detected using Nelore genotyping data

The number of SNPs in each detected CNV varied from 20 to 1,420 (94 ± 110). CNV length varied from 20.01 Kb to 7.75 Mbp (320 ± 413 Kbp). Figure 2 (Additional file 1) shows the size distribution of detected CNVs. Observed CNVs larger than the average by one standard deviation or more (733 Kbp) and with a frequency greater than 1 % were rare and far apart (*n* = 116), with a mean frequency of 2.47 %.

### CNVR identification

**Fig. 3:**
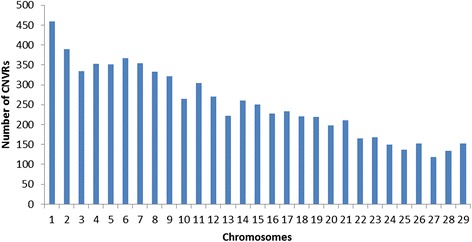
Distribution of CNVRs detected using Nelore SNP genotyping data across bovine chromosomes

**Fig. 4:**
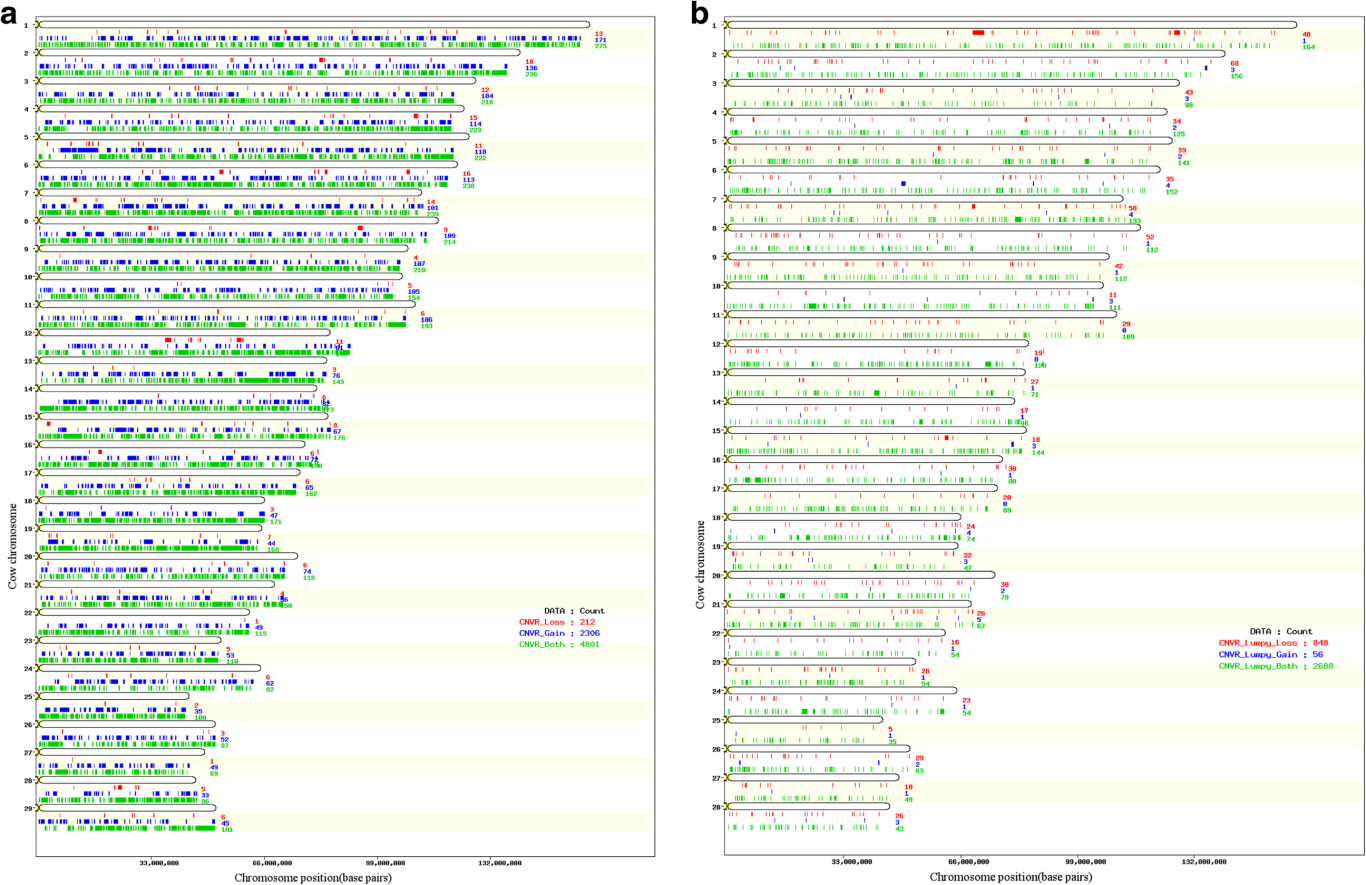
Distribution of gain, loss and mixed CNVRs detected across the Nelore genome (based on UMD3.1). **a** CNVRs detected with genotyping data. **b** CNVRs <5 Mb detected with NGS data

Different methods for condensing overlapping CNVs into Copy Number Variant Regions (CNVRs) have been proposed [45–47]. Nelore CNVRs were identified using JM-CNV [48], which considers CNV length and frequency, removes extremely long or infrequent CNVs from the initial analysis, and resolves observed breakpoint issues [24, 25]. The 68,007 detected CNVs were condensed into 7,319 CNVRs (Fig. 3: Additional file 2), representing a total coverage of 1.56 Gigabases (61.91 %) of the bovine autosomal genome (Additional file 3). A total of 2,306 duplications, 212 deletions, and 4,801 duplications and deletions were observed in the identified CNVRs (Fig. 4a). A high positive correlation between the number of detected CNVRs and the size of bovine chromosomes was observed (0.98, Fig. 3), contrary to what was observed in terms of the number of total CNVs detected (0.34, Fig. 1).

**Fig. 5:**
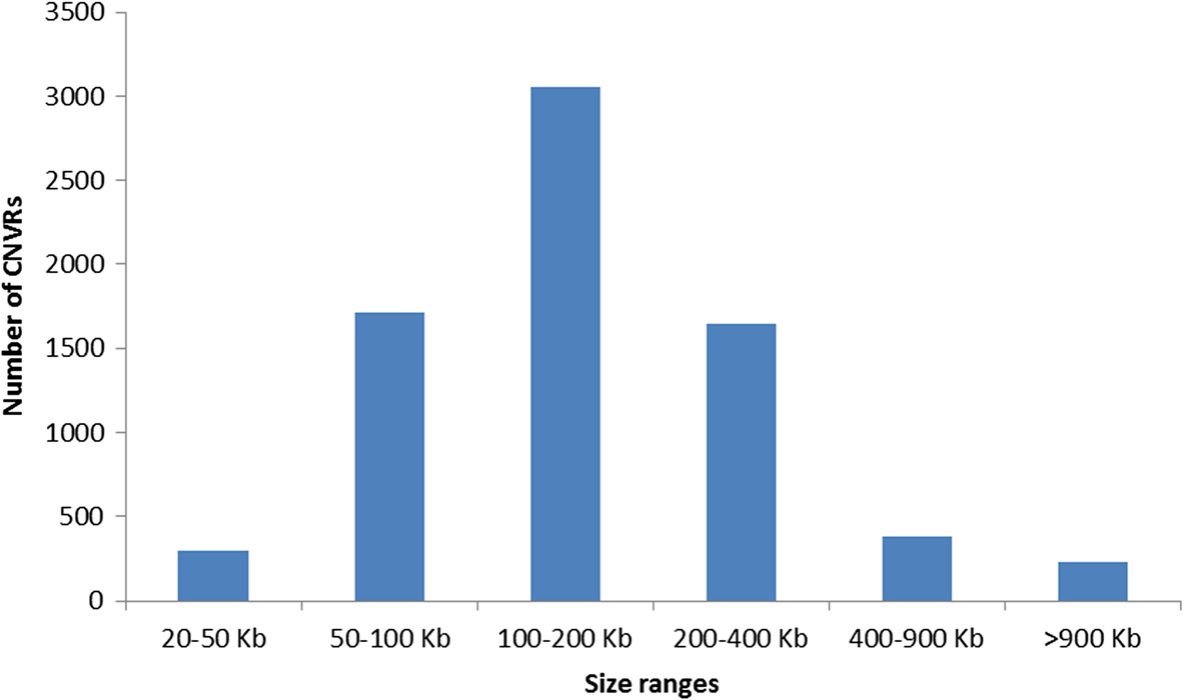
Size distribution of CNVRs detected using Nelore genotyping data

CNVR length varied from 20.1 Kbp to 3.81 Mbp (213 ± 237 Kbp, Fig. 5, Additional file 2). BTA1 was found to have the highest number of CNVRs (459), while BTA27 had the lowest number (119) of CNVRs (Additional file 2). As for the average distance between CNVRs, BTA24 and BTA19 were found to have the greatest (444.8Kbp) and the smallest (323.5Kbp) distances, respectively. A total of 962, 713, and 296 CNVRs showed frequencies >1, >2 and >10 % in the studied samples, respectively.

### CNVs in NGS data

**Fig. 6:**
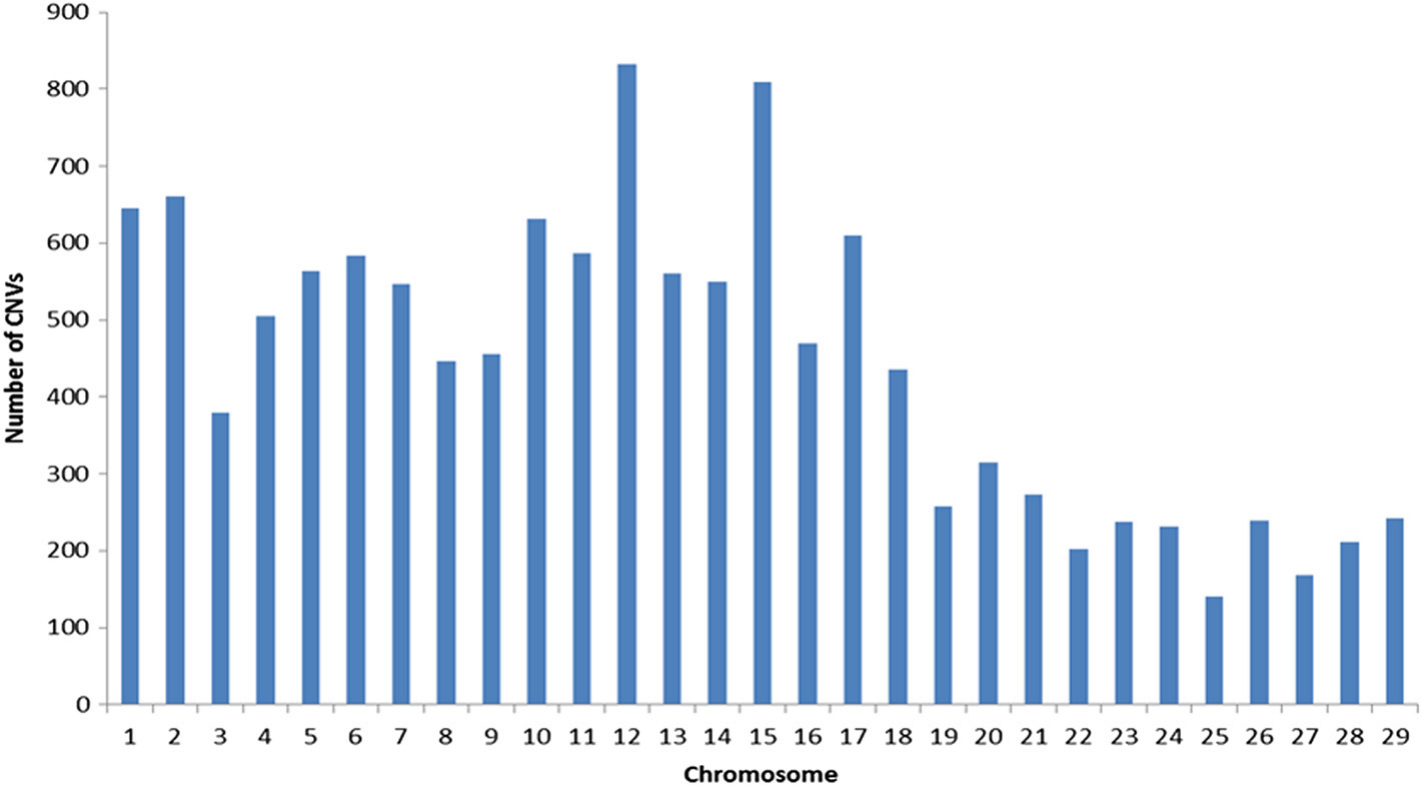
Chromosome distribution of CNVs detected using Nelore NGS data

LUMPY [49] uses signal depth from observed split-reads and from miss-mapped paired-end reads as evidence to identify CNVs. A total of 12,786 CNVs distributed non-uniformly (Fig. 6) along the 29 autosomes, representing 999 duplications and 11,787 deletions, with average sizes of 252.8 ± 692.0 Kbp and 22.9 ± 194.2 Kbp, respectively, were detected in NGS data from eight resequenced bulls when both types of evidence were considered (Additional file 4).

Even though the analyzed NGS dataset was exceedingly smaller than the SNP dataset (8 vs 1,509 animals), and represents a reduced sample of the breed’s genetic diversity, LUMPY detected more than ten times the number of CNVs detected with PennCNV, when the same eight animals were considered. Similar results have been reported in other studies [35, 50] and may be attributed to the better resolution of CNV breakpoints which can be obtained from NGS data. Moreover, the CNV ratio of deletions to duplications observed in the results obtained from NGS data (11.80) is more than 56 times larger than the ratio obtained from genotyping data (0.21), suggesting the method is more sensitive in identifying deletions. JM-CNV [48] was used to converge identified CNVs >1,000 bp into CNVRs. The 12,786 detected CNVs were condensed into 3,781 CNVRs, representing a total of 84 duplications, 909 deletions, and 2,788 duplications and deletions (Fig. 4b, Additional file 5). Inevitable ascertainment bias may have influenced obtained results, as the reference bovine genome sequence was derived from a Hereford individual (*Bos taurus*). Future analysis may be used to identify and correct this when a reliable *Bos indicus* reference sequence becomes available.

### CNV and CNVR independent validation and cross-referencing

The importance of comparing CNV detection results with complementary techniques, such as qPCR, FISH, CGH arrays, SNP arrays, and sequencing has been extensively reviewed in cattle [35, 51]. Cross-validation of CNVs detected in the genotyping data was performed with NGS data from the eight resequenced animals. A total of 988 CNVs were detected with genotyping data from the eight animals (Additional file 6) and 909 (92 %) of these overlapped with 50 bp or more of at least one of 57,968 CNVs identified with LUMPY using evidence from split-reads and/or miss-mapped paired-end reads - Table 1 (see Additional file 7 for complete list). Further evaluation of the 909 CNVs identified using SNP and NGS data revealed that 173 were identified with all three independent types of evidence (SNP data and signal depth from observed split-reads and from miss-mapped paired-end reads), while 736 were identified with at least two types of evidence (SNP data and observed split-reads or miss-mapped paired-end reads).

**Table 1:** Summary of CNVs detected using SNP and resequencing data

Animal	# of CNVs detected in SNP data	# of CNVs validated with NGS data	% Validated
BINE_01	11	4	36
BINE_02	87	86	99
BINE_03	27	14	52
BINE_04	22	22	100
BINE_05	93	93	100
BINE_06	729	672	92
BINE_07	9	9	100
BINE_08	10	9	90
Total	988	909	92

**Fig. 7:**
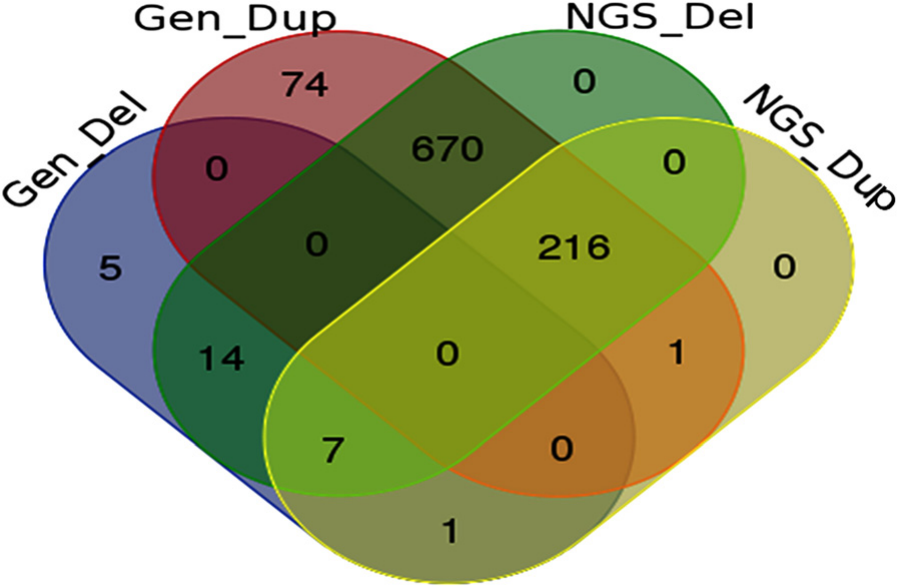
Number of non-redundant CNVs (Dup = Duplications and Del = Deletions) detected using genotyping and NGS data

**Fig. 8:**
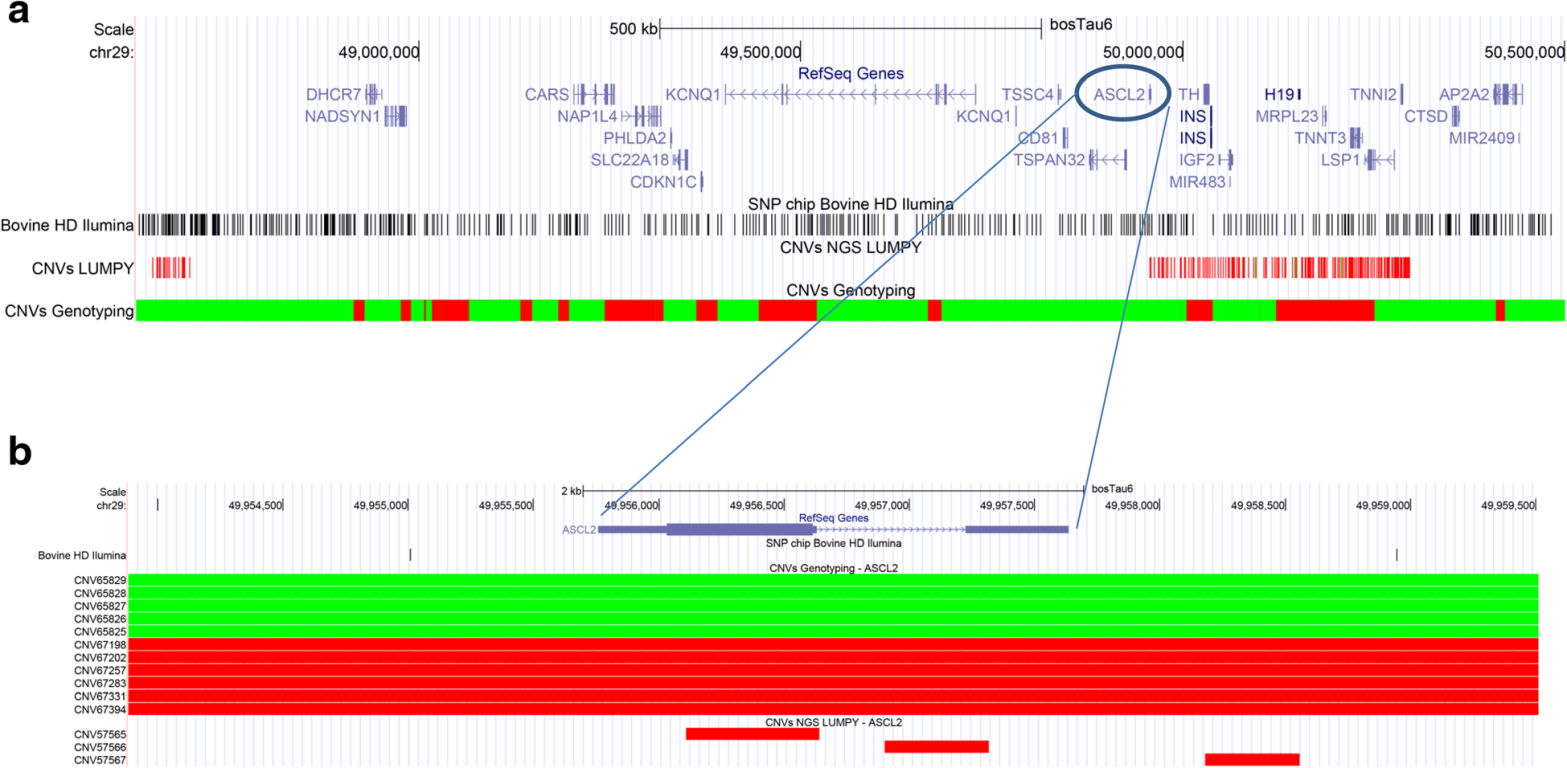
Cross-comparison of CNVs detected with SNP and NGS data. (A) Chromosomal region (BTA29:48,630,000–50,500,224) with detected duplication (green) and deletion (red) CNVs. (B) CNVs intersecting the ASCL2 gene

A total of 886 of the 988 CNVs (90 %) were observed to contain mixed segments of duplications or deletions considering mostly the NGS data (Fig. 7), which should be considered in future studies as complexity negatively correlates with reproducibility in subsequent CNV studies with different platforms [52]. The high proportion of observed cross-validated CNVs was contrasted with results reported by previous studies [52, 53]. Observed results show that some CNVs detected with genotyping data overlap with multiple smaller CNVs detected with NGS data (Fig. 8), confirming previous reports [26, 52, 54] which show that NGS offers higher resolution and precision for identification of CNV boundaries.

A total of 68,007 CNVs identified with the SNP dataset were cross-matched with 179 CNVRs previously validated with at least two distinct methods available at DVGarcheive database (http://www.ebi.ac.uk/dgva/data-download) and in the literature [26, 27, 30, 33]. A total of 62 % (111) of previously validated cattle CNVRs were found to overlap with CNVs identified in Nelore cattle, considering a minimum of 10 kb of overlap [55, 56]. CNVs with frequencies >1 % were observed in 41 of these previously reported CNVRs in the analyzed Nelore samples (Additional file 8).

Bickhart et al. [26] reported 730 Nelore CNVs from analyses of NGS data from a single animal, considering BTAU4.0 as reference assembly. Conversion of BTAU4.0 to UMD3.1 coordinates using Liftover [57] resulted in 458 CNVs and a total of 295 (64.4 %) of these were found to overlap with one or more of the CNVs currently identified in the NGS data. Observed discrepancies may have resulted from specificities of applied methods as well as sampling bias caused by the extremely reduced sample size used by [26].

### CNVRs in regions containing QTLs in cattle

Recent studies [27, 30, 36, 37] revealed CNV variants associated with production traits in dairy and beef cattle. Reported findings suggest that models combining SNP and CNV data could be more powerful at capturing the underlying variation and therefore provide more accurate frameworks to better account for the heritability of complex traits, as the effect of 25 % of identified CNVs could not be accounted for by neighboring SNPs [27].

CNVRs have been detected in genomic regions shown to contain cattle QTLs and have been shown to affect body measurements [17], production traits [37] and parasite resistance [30]. The 7,319 CNVRs detected with genotyping data were compared to the 11,506 regions of the bovine genome reported to contain QTLs (QTL database http://www.animalgenome.org/cgi-bin/QTLdb/BT/index). A total of 9.4 % (688/7,319) of the detected CNVRs, which encompass a total of 312Mbp of the bovine autosomal genome, were observed to overlap by >50 % [17] of 286 non-redundant QTLs associated with economically important production traits such as residual feed intake, gestation length, marbling score, fat thickness at the twelfth rib, dry matter intake, longissimus muscle area, clinical mastitis, and carcass weight (Additional file 9).

**Fig. 9:**
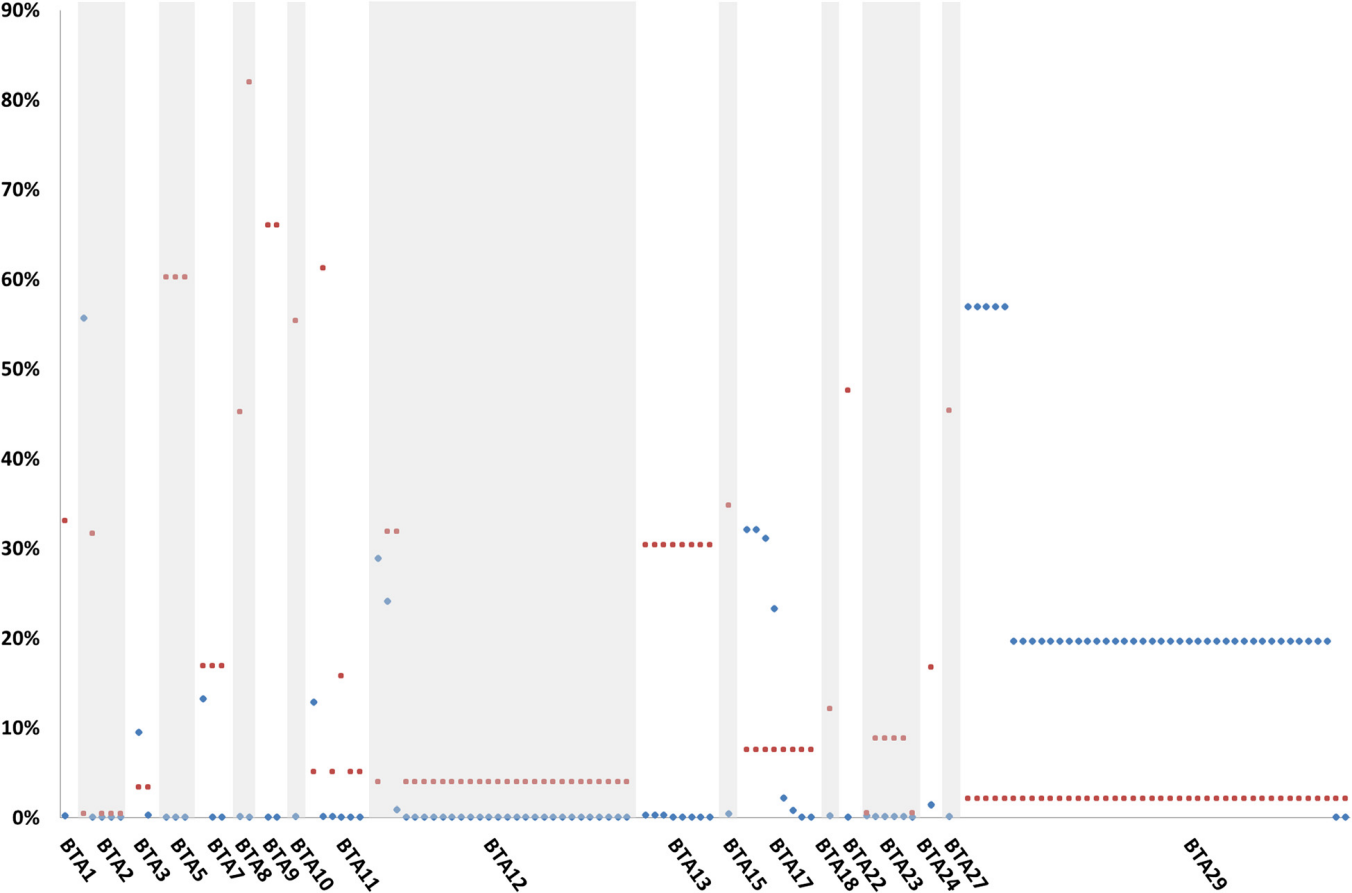
Chromosome distribution of relative CNV estimated frequencies in Nelore (blue) and Holstein (red) cattle [37]

All of the 34 CNVs found by [37] to be associated with milk production traits in Holsteins (HOL) were also observed in Nelore (NEL) cattle (Additional file 10). Comparisons of estimated frequencies of these CNVs in the two breeds revealed 14, 13, 6 and 14 regions in high (>20 % in both breeds), low (<20 % in both breeds) and divergent (NEL > HOL, NEL < HOL) frequencies, respectively. Figure 9 shows chromosome positions and frequency differences between Nelore and Holstein cattle at these CNVs.

Considering the distinct selective pressures Nelore and Holstein cattle have been historically under either naturally (tropical versus temperate climates) or artificially (beef versus milk production), frequency deviations are expected in underlying variant regions controlling traits under selection. CNVR_7294 was observed in 56.93 % of the Nelore samples tested, while a CNV located in the same position at frequency of 2.09 % was reported to be strongly associated with protein percentage in Holsteins (FDR = 5,09E-05 [37]). This genome region harbors QTL controlling carcass weight (QTL 13550), milk fat percentage (QTL 13547) and Milk protein percentage (QTL 13548), and the observed frequencies suggest the CNV may be under positive selection in Nelore while strong negative selection in Holsteins. A similar pattern of frequency divergence can be observed with CNVR_7295. Conversely, CNVR_1557, CNVR_3011 and CNVR_4292, located in regions reported to contain QTL affecting beef production traits, were observed at low frequencies in Nelore cattle (0.07 %) and at high frequencies in Holsteins (60.26, 66.05 and 30.42 %, respectively), suggesting these CNVs may contribute to the underlying variation in traits under divergent selection in these breeds. These observations suggest that more extensive studies with CNV data from divergent breeds or other population structures could help identify signatures of selection in genome regions containing segmental variations.

### Gene ontology and CNVRs

The occurrence of CNVs in genome regions containing functional genes may create opportunities for the emergence of new allelic variants, gene isoforms, and complex mechanisms of gene expression control as a consequence of naturally occurring evolutionary processes. A total of 4,097 CNVRs (55.98 %) are located within genome regions containing 10,399 annotated genes, which can be functionally classified as protein coding (*n* = 10,070), microRNA (*n* = 159), snoRNA (*n* = 148), snRNA (*n* = 10), miscRNA (*n* = 8), and rRNA (*n* = 2).

**Fig. 10:**
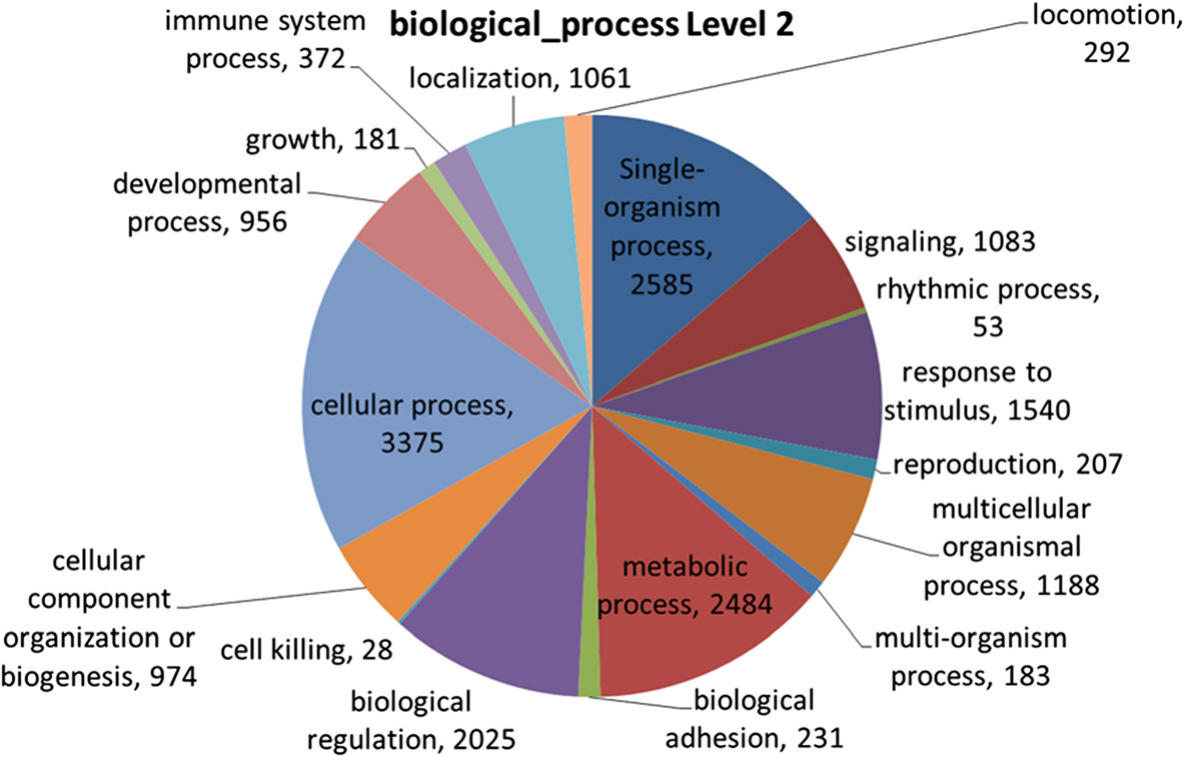
GO annotation for biological processes of CNVs detected in Nelore cattle

Automated annotation of these genes with GO terms revealed important categories, including metabolic and cellular processes, biological regulation, response to stimulus, cell signaling, reproduction, and growth (Fig. 10). Many well described contrasting traits between taurine and zebu cattle have been targets of natural selection and production-oriented genetic improvement, and are mediated by genes involved in these biological processes, including reproduction (age of first estrous, fertility, calving interval, etc.) [58], resistance to endo- and ectoparasites [59], heat tolerance [60], disease resistance [61], as well as growth and carcass and meat quality traits [62]. Therefore, further investigation of these regions may unveil important information for understanding underlying mechanisms affecting economically important traits.

Previous studies to identify CNVs in cattle using small numbers of samples from divergent breeds have focused specially on comparisons between breeds [26] and may have provided a comprehensive view of breed-specific CNVs potentially associated with contrasting traits observed among evaluted breeds. Analysis of 1,509 Nelore samples allowed a broad identification of CNVs segregating within the breed in addition to generating population frequency estimates and therefore providing crucial information for inference if observed CNVs may indeed be under selection within the breed. Several previously reported CNVs [26, 63–65] within genome regions containing genes that may control traits of interest for cattle production were observed at extremely low frequencies in the population studied herein (Additional file 10), indicating that these variants may not be positively associated with underlying factors associated with traits under positive selection in the breed.

Sequencing of the bovine reference genome revealed the expansion of the antimicrobial cathelicidin gene, found as a single copy in humans and mice, into a large gene family in cattle [66] . Bickhart et al. [26] reported that one of these cathelicidin genes (CATHL4) was observed to be highly duplicated in the single evaluated Nelore sample. A single copy duplication spanning this gene was observed in both SNP and NGS data but at frequencies <1 %, indicating this particular CNV is not undergoing strong positive selection in the breed (Additional files 2 and 11). Similar divergent results were observed with other genes previously reported to be located in genome regions with CNVs in Nelore cattle and that have been independently shown to affect height (*pleiomorphic adenoma gene 1* - PLAG1), lipid metabolism (*apolipoprotein L3* - APOL3 and *sterol carrier protein 2* - SCP2), transport (*fatty acid binding protein 2* - FABP2, *vesicle associated membrane protein 7* - VAMP7, *lecithin-cholesterol acyltransferase* - LCAT, and *lecithin-cholesterol acyltransferase* - PCTP), endoparasite resistance (*UL16-binding protein 17* - ULBP17), and oxidative metabolism *(aldehyde oxidase 1* - AOX1) (Additional files 2 and 11).

Genetic imprinting represents a major mechanism of epigenetic regulation of gene expression leading to parent-specific differential expression of a subset of 20 bovine genes (Imprinted Gene Databases - http://www.geneimprint.com/site/genes-by-species.Bos+taurus [67]) and DNA sequence polymorphisms in imprinted genes have been shown to affect production traits in cattle [68]. CNVs were observed in regions spanning 11 imprinted genes in Nelore cattle: *mesoderm specific transcript* - MEST (BTA4), *nucleosome assembly protein 1 like 5* - NAP1L5 (BTA6), *insulin like growth factor 2 receptor* - IGF2R (BTA9), *neuronatin* - NNAT (BTA13), *antisense transcript gene of PEG3* - APEG3 (BTA18), *maternally expressed 3* - MEG3 (BTA21), *pleckstrin homology like domain family A member 2* - PHLDA2 (BTA29), *tumor-suppressing subchromosomal transferable fragment 4* - TSSC4 (BTA29), *achaete-scute family bHLH transcription factor 2* - ASCL2 (BTA29), *insulin like growth factor 2* - IGF2 (BTA29), and H19 (BTA29) (Additional file 11).

Observed CNV frequencies in regions harboring MEST, NAP1L5, IGF2R, NNAT, APEG3, and MEG3 were very low (<0.2 %). Conversely, CNV frequencies in the region with imprinted genes on BTA29 (49,329,504-50,163,147 bp) were greater than 9 %. The PHLDA2 gene (also known as TSSC3) is located in the aforementioned region of BTA29 and is expressed in the bovine placenta and embryonic tissues during pregnancy [69, 70]. Comparisons of bovine and human polypeptides revealed a strong homology and suggested that PHLDA2 could be involved in the same regulatory pathways in both species [69]. According to Huang et al. [71], proper PHLDA2 expression is essential for normal embryo development during early development. Additional studies show that PHLDA2 may affect the development of bovine pre-implantation embryos [72]. A single copy duplication in the region containing PHLDA2 was observed in a total of 128 individuals (Additional files 2 and 11) and should be considered in future studies to evaluate the effect of this gene in early embryo development.

### Annotation of most frequent CNVRs in Nelore cattle

**Fig. 11:**
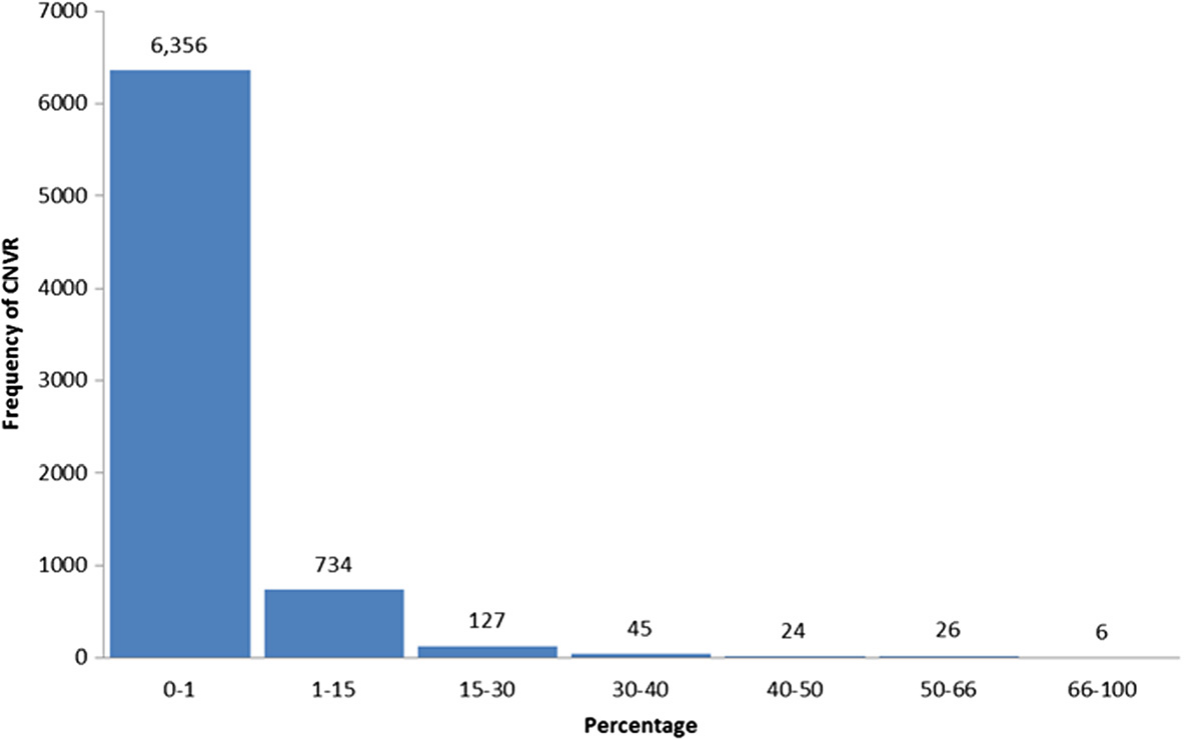
Frequency distribution of CNVRs detected using SNP genotyping data from a population of 1,509 Nelore cattle

CNVs with frequencies higher than 1 % were observed in a total of 13 % (962/7,319) of the detected CNVRs (Fig. 11). Six CNVRs were observed to be highly frequent in Nelore, with more than 1,000 CNVs in the analyzed samples and may therefore be associated with underlying factors positively affecting traits under selection in the breed.

BTA2:104,853,165-105,006,347 contains a duplication that was observed in a total of 1,056 individuals. This genome region harbors genes such as *insulin-like growth factor binding protein 2* (IGFBP2) and *short stature homeobox* (SHOX), among others. Studies in humans show that mutations in this gene can lead to short stature and to different pathological conditions such as Turner syndrome (TS), Léri-Weill dyschondrosteosis, and Langer mesomelic dysplasia [73–76]. IGFBP2 has also been shown to be involved in regulating the estrous cycle and early pregnancy in cattle [77].

BTA4:114,375,180–114,638,146 contains 16 annotated genes, as well as microRNA 671, and was found to be duplicated in more than 1,000 animals and one resequenced individual, and to be deleted in four genotyped animals. Studies on humans show that *cyclin-dependent kinase 5* (CDK5), which is located in this region, plays an important role in central nervous system function. It has also been proposed that CDK5 is important in myogensis, hematopoietic cell differentiation, spermatogenesis, insulin secretion, and lens differentiation [78, 79]. A study with pigs showed that CDK5 is involved in brain development [80].

BTA6:119,154,914–119,384,691 contains *actin binding LIM protein family member 2* (ABLIM2), *actin filament associated protein 1* (AFAP1), *sortilin related VPS10 domain containing receptor 2* (SORCS2), *prosaposin-like 1* (PSAPL1), and *SH3 domain and tetratricopeptide repeats 1* (SH3TC1). This CNVR was found to be duplicated in more than 1,000 animals and deleted in 20 animals. Klimov et al. showed that the ABLIM2 protein is necessary for normal neuron functioning [81]. SORCS2 was identified as a proneurotrophin receptor and is expressed as a single-chain protein that is essential for proBDNF-induced growth cane collapse in developing dopaminergic processes. Deficiency of SORCS2 in mice caused reduced dopamine levels and metabolism, and dopaminergic hyperinnervation of the frontal cortex [82, 83].

BTA19:48,427,331–48,537,167 harbors *angiotensin I converting enzyme* (ACE), *WD40 repeat-containing protein* (WDR68), and *potassium voltage-gated channel subfamily H member 6* (KCNH6). The ACE gene encodes an enzyme involved in catalyzing the conversion of angiotensin I into angiotensin II, which is a potent vasopressor that controls blood pressure and fluid-electrolyte balance. Gauthier et al. (2013) demonstrated that ACE inhibitor-enhanced bradykinin relaxations of bovine coronary arteries occurs through endothelial cell B1 receptor activation and nitric oxide [84].

BTA19:63,507,097–63,735,382 *contains protein kinase C alpha* (PRKCA), *calcium voltage gated channel auxiliary subunit gamma 4* (CACNG4), and *calcium voltage-gated channel auxiliary subunit gamma 5* (CACNG5) genes, as well as the 7SK misc-RNA and was found to be duplicated in more than 1,000 animals and deleted in 11 animals in the population studied. A study on cattle showed that 7SK misc-RNA is located on a central region of the *hexamethylene bis-acetamide inducible 1* (BHEXIM1) gene and may play an important role in gene regulation [85]. The authors proposed that this gene affects the latent life cycle of the bovine immunodeficiency virus (BIV), which leads to a lack of clinical signs of the disease in affected animals. This region may be of interest for studies on the clinical diagnosis and prevention of this disease.

## Conclusions

This study represents the first comprehensive CNV survey within the Nelore breed (1,717 animals and ~770 K SNPs). Obtained results allowed for direct comparisons of CNV detection results with two distinct platforms (HD SNP genotyping and NG sequencing), and with previous reports from independent studies.

The bovine CNV map was significantly enriched, particularly for the Nelore breed and associated variant frequency estimates enabled the identification of variants potentially associated with traits under selection, particularly in genome regions harboring QTLs affecting production traits.

Obtained results suggest that more extensive studies using CNV data from divergent breeds with differing population structures could help identify signatures of selection using approaches frequently used with SNP data. The study provides important information that may inspire or contribute to future studies on the association between CNVs and production traits important for genetic improvement in cattle.

## Methods

### Animals

DNA was extracted from commercially available semen samples, and from hair and venous blood samples obtained from animals in production farms, as part of routine animal handling and testing procedures. Tissues were processed with standard commercial kits.

### Genotyping and resequencing data

A total of 1,717 Nelore (*Bos indicus*) samples were genotyped with the Illumina Bovine HD Genotyping Bead Chip. DNA was extracted from semen, blood, or hair samples from registered and production animals from commercial farms in Brazil. In addition, DNA from eight unrelated Nelore founding bulls was resequenced using Illumina HiSeq2000 paired-end reads with a minimum coverage of 20× (Table 2) [86].

**Table 2:** Genome coverage of eight resequenced animals

Animal ID	Length (bp)	# Aligned	# Unaligned	Total reads	mapped reads	Seq. X coverage
BINE_01	2512082506	1343863053	48758778	1392621831	96.50 %	53
BINE_02	2512082506	1301229539	15069073	1316298612	98.86 %	52
BINE_03	2512082506	1587268578	17505393	1604773971	98.91 %	63
BINE_04	2512082506	645452383	40423110	685875493	94.11 %	26
BINE_05	2512082506	554921159	5980155	560901314	98.93 %	22
BINE_06	2512082506	554580777	6327041	560907818	98.87 %	22
BINE_07	2512082506	1399071315	16203693	1415275008	98.86 %	56
BINE_08	2512082506	639537955	7497809	647035764	98.84 %	25

### CNV and CNVR detection in genotyping data

Illumina genotyping data was analyzed with PennCNV [87]. Log R Ratios (LRR), B Allele Frequencies (BAF), distances between neighboring SNPs, and pedigree information were used by the Hidden Markov Model (HMM) algorithm to detect CNVs. Only autosomal SNPs were considered in the analysis. Initial analysis of the dataset with default LRR and BAF cut-off values normally used in CNV studies on humans [88, 89] resulted in the exclusion of 997 animals (data not shown). Adjusted LRR and BAF cutoff values were derived for analysis of the Nelore dataset based on the observed distributions of these variables in the studied samples. New LRR and BAF cut off values were identified to independently exclude 10 % of the samples. In addition, a GC content correction was performed for each SNP in regions located 500Kb upstream and downstream from each studied SNP [32]. Use of new LRR (<0.4) and BAF (<0.04) cut-off values in conjunction resulted in removal of 208 samples (12 %) from the final dataset. PennCNV default procedures and parameters were subsequently used in the analysis.

Overlapping CNVs were grouped into CNVRs using JM-CNV [48]. CNVs were grouped into closed intervals of whole numbers. This choice made CNVR definition more natural and included the set of intervals whose overlap did not exceed the average size of the CNV set plus one standard deviation. Meanwhile, long and infrequent CNVRs were grouped separately so they would not skew estimated averages and standard deviations.

### CNV detection in NGS data

A previously described strategy for determining high-resolution CNVs in humans [90] was used to identify CNVs in Illumina shotgun data from eight key ancestral Nelore bulls. Paired-end reads were mapped onto the UMD 3.1 assembly using BWA with default parameters [91]. CNVs were detected using LUMPY, a novel CNV discovery framework that uses multiple detection signals including read depth from split reads and mis-mapped paired ends [49] (Additional file 4) for CNV identification. Only autosomal regions were considered in the analysis. Overlapping CNVs >1,000 bp were grouped into CNVRs using JM-CNV [48].

### Cross validation of CNVs

CNVs detected with SNP genotyping data were cross-validated using a combination of information derived from eight resequenced Nelore bulls and from published literature, following previously reported strategies [52, 56]. Sequence coordinates from CNVs detected using genotyping methods (Additional file 8) were initially compared to coordinates from 179 CNVRs previously validated in independent studies [26, 27, 30, 92]. Coordinates from CNVs observed with PennCNV and LUMPY were compared using a script written in Python [53] (Additional files 6 and 7). All CNVs >50 bp detected with LUMPY were used in this procedure.

### Functional annotation

Automated annotation of genes present within observed CNVs was performed using the scan_region.pl tool from PennCNV and the annotation file of UMD3.1 assembly [93]. Ensembl Genes 77 database (*Bos taurus* genes UMD3.1) and BioMart were used to annotate observed CNVRs. FASTA sequence files containing annotated gene regions from observed CNVRs were imported into Blast2GO [94, 95] for automatic functional annotation. These files were blasted against the NCBI nr database using default BlastX parameters (e-value threshold 1e-03 and HSP length cut-off of 100). Sequence mapping for Gene Ontology (GO) terms was performed using default parameters (e-value hit filter of 1e-06, annotation cut-off of 55, and GO weight of 5). Annotations were performed using the Annex function of the GO Annotation Toolbox [96]. InterProScan terms were obtained following a previously reported method [97]. In addition, metabolic pathway maps were obtained using the method outlined by the KEEG PATHWAY database [98]. Overlaps between detected CNVs and CNVRs and previously detected QTLs from the Bovine QTL Database [99] were identified with a script in Python (Additional file 9).

## Additional files

Additional file 1: CNVs detected in Nelore HD SNP genotyping data. (XLSX 48 kb) Additional file 2: CNVRs detected in Nelore HD SNP genotyping data. (XLSX 1160 kb) Additional file 3: Descriptive statistics of CNVRs per individual bovine chromosome. (XLSX 5510 kb) Additional file 4: CNVs detected in Nelore NGS Data. (XLSX 450 kb) Additional file 5: CNVRs detected in Nelore NGS Data. (XLSX 12 kb) Additional file 6: CNVs detected in HD SNP genotyping data from the eight resequenced bulls. (XLSX 648 kb) Additional file 7: Overlap of CNVs detected with both genotyping and NGS data from eight key ancestral bulls. (XLSX 196 kb) Additional file 8: Overlap of all CNVs identified in Nelore cattle with CNVRs currently listed at DVGarcheive database. (XLSX 57 kb) Additional file 9: CNVRs overlapping with previously detected QTLs from the Bovine QTL Database. (XLSX 2973 kb) Additional file 10: CNVs reported by Xu et. al (2014a) to be associated with milk production traits in Holsteins also observed in Nelore cattle. (XLSX 289 kb) Additional file 11: Gene Ontology annotation in CNVRs detected in Nelore HD SNP genotyping data. (XLSX 313 kb)
